# Relationship between the Developmental Coordination Disorder Questionnaire 2007 and the Bruininks-Oseretsky Test of Motor Proficiency Second Edition in Korean Children

**DOI:** 10.3390/children9020255

**Published:** 2022-02-14

**Authors:** Deukgeun Yoon, Misun Kim, Seokyeon Ji, Dabin Choi, Yoo-Sook Joung, Eun Young Kim

**Affiliations:** 1 Department of Occupational Therapy, Soonchunhyang University, Asan-si 31538, Korea; emrrms9208@gmail.com; 2 Center of Sensory Integration toward Social and Occupational Being, Seoul 04061, Korea; meesun89@gmail.com (M.K.); jiseokyeon@gmail.com (S.J.); 3 Department of ICT Convergence, Soonchunhyang University, Asan-si 31538, Korea; choidabin08@gmail.com; 4 Department of Psychiatry, Samsung Medical Center, Sungkyunkwan University School of Medicine, Seoul 06351, Korea

**Keywords:** motor assessment, motor skills, developmental coordination disorder, Korean children

## Abstract

This study investigated the relationship between the Developmental Coordination Disorder Questionnaire 2007 (DCDQ’07) and the Bruininks-Oseretsky Test of Motor Proficiency Second Edition (BOT-2) in Korea. This study also adjusted the cutoff score of the DCDQ’07 based on the BOT-2 for Korean children. A total of 256 children were recruited from communities in Korea. They were divided into two age groups: 8 to 9 years old and 10 to 12 years old. Children performed the BOT-2, and their parents completed the DCDQ’07. The correlation between the DCDQ’07 and the BOT-2 was analyzed. The adjusted DCDQ’07 cutoff score for Korean children was calculated using the BOT-2 as the criterion through a receiver operating characteristic curve. A significant correlation between the DCDQ’07 and the BOT-2 was found, indicating that Korean parents’ perception of children’s motor skills was related to their children’s actual motor proficiency. The adjusted cutoff score of the DCDQ’07 had a sensitivity of 72.7–85.7% and a specificity of 62.5–64.0%. This study demonstrated that children’s motor skills reported by Korean parents on the DCDQ’07 were valid based on a community sample. The adjusted cutoff score of the DCDQ’07 could be used to identify children suspected of having a developmental coordination disorder.

## 1. Introduction

Children with developmental coordination disorder (DCD) have difficulties in coordinated motor skills affecting daily activities [1]. The prevalence of DCD is 5 to 6% in school-aged children [2], which means that one out of 20 students in a class would have motor skill deficits. DCD has a high comorbidity with other neurodevelopmental disorders [3]. About half of children with attention-deficit/hyperactivity disorder or specific learning disorder have DCD [4,5]. DCD is linked to not only motor impairments but also various problems in other domains [6,7]. Children with DCD are more likely to have low self-efficacy [8], emotional symptoms such as depression and anxiety [9,10], cognitive dysfunction [11], poor social skills [12], and challenges in school performance [12,13]. These problems with DCD can compromise their participation in life situations [14] and quality of life [7]. Moreover, the impact of DCD can persist to later developmental stages [15,16]. Thus, identifying children with DCD at the appropriate time is necessary for timely intervention and the prevention of secondary issues [17].

The diagnosis of DCD is based on the assessment of a child’s motor proficiency and motor skills in daily life [18]. To determine whether a child’s motor skills are markedly below expectations for the child’s age, a standardized movement test can be used. Representative movement tests include the Movement Assessment Battery for Children–Second Edition (MABC-2) [19] and the Bruininks-Oseretsky Test of Motor Proficiency Second Edition (BOT-2) [20]. The MABC-2 can efficiently assess motor ability to identify children with motor impairment in a relatively short time. The BOT-2 comprehensively measures overall motor ability to understand a child’s motor proficiency profile as well as motor deficits. The BOT-2 is the most widely used standardized assessment of children’s motor competence by occupational therapists in Korea [21,22]. To examine whether poor motor skills cause difficulties in a child’s daily life, the Developmental Coordination Disorder Questionnaire 2007 (DCDQ’07) [23] can be used. It asks parents how well their child is doing in daily activities that demand motor skills. These motor assessments support the DCD diagnostic process.

Standardized performance-based tests such as the BOT-2 can objectively measure an individual’s ability with structured administration. In contrast, questionnaires such as the DCDQ’07 can assess a child’s motor skills through the perception of a respondent (e.g., the parents). The respondent can provide meaningful information relevant to daily activities. However, such information might be subjective. Although the parents’ perception of their children’s motor skills measured through the questionnaire was basically related to the children’s actual motor ability evaluated by the performance-based test [24], the strength of such relationship varied across studies [25,26]. Three factors (control during movement, fine motor/handwriting, and general coordination) of the DCDQ’07 were related to all four motor areas (fine motor control, manual coordination, body coordination, and strength and agility) of the BOT-2 in Brazil [27], whereas only the gross motor of the DCDQ-parent, the previous version [28] of the DCDQ’07, was significantly correlated with manual coordination of the BOT-2 in Australia [29]. Such inconsistency between previous findings might be attributed to differences in parents’ perspectives across cultures. The present study examined the relationship between the DCDQ’07 and the BOT-2 to understand how well Korean parents’ perceptions of their children’s motor skills corresponded to their children’s actual motor proficiency.

The DCDQ’07 assists in identifying children with DCD using a cutoff score established with Canadian and British children [23]. The cutoff score needs to be validated or adjusted when the DCDQ’07 is adapted to other cultures [30,31]. Lower cutoff scores have been proposed for Indian children [30] and Brazilian children aged over 8 years [31]. Given that there are differences in parents’ ratings of their children between cultures, the DCDQ’07 cutoff score needs to be investigated in Korea. Thus, another objective of this study was to examine whether the original cutoff score could successfully identify children with or without probable DCD and whether the adjusted cutoff score could be more precisely applied to Korean children.

## 2. Materials and Methods

### 2.1. Participants and Procedure

A total of 256 Korean children aged 8 to 12 years (mean age, 10.24 years, SD = 1.51; 128 boys, 128 girls) and their caregivers were included in this study. There were 59 eight-year-olds, 75 nine-year-olds, 49 ten-year-olds, 17 eleven-year-olds, and 56 twelve-year-olds. They were divided into two age groups: 8–9 years old (mean age, 9.05 years, SD = 0.60; 67 boys and 67 girls) and 10–12 years old (mean age, 11.55 years, SD = 1.04; 61 boys and 61 girls). These participants were recruited from communities of Gyeonggi-do (*n* = 125), Seoul (*n* = 99), Chungcheong-do (*n* = 23), Daejeon-si (*n* = 7), and Gwangju-si (*n* = 2), Korea. This study was based on community samples. It also included children with developmental coordination disorder (*n* = 1), attention-deficit/hyperactivity disorder (*n* = 4), specific learning disorder (*n* = 1), autism spectrum disorder (*n* = 1), anxiety disorder (*n* = 1), or language disorder (*n* = 1), reported by parents. Children who had medical conditions (e.g., fracture) or those who did not complete the assessments were excluded.

A cross-sectional design was used in this study. This study was part of the Korean BOT-2 standardization project, which was approved by the Ethics Committee of Soonchunhyang University. Informed consent was provided by all participants. Data collection was conducted from November 2019 to July 2021 in community centers, schools, and university settings. BOT-2 examiners were 28 occupational therapists with a mean testing experience of 33. One examiner administered all fifty-three BOT-2 items to 106 children. Two or more examiners split the BOT-2 items between themselves and administered the items to 150 children. Children could take a break when they wanted. Most children performed the BOT-2 in one day, except three children were tested on two days at a 10-day interval. Children’s parents completed the DCDQ’07 at an average of 4 days prior to BOT-2 administration.

### 2.2. Measures

#### 2.2.1. The Bruininks−Oseretsky Test of Motor Proficiency Second Edition (BOT-2)

The BOT-2 is a norm-referenced and standardized test that measures overall motor ability of individuals aged 4 to 21 years [20]. An examinee is asked to perform each item according to the instruction of an examiner. The BOT-2 has eight subtests comprised of 53 items: seven items on fine motor precision (e.g., cutting out a circle), eight items on fine motor integration (e.g., copying a square), five items on manual dexterity (e.g., placing pegs into a pegboard), seven items on bilateral coordination (e.g., jumping jacks), nine items on balance (e.g., standing on one leg on a line), five items on running speed and agility (e.g., one-legged side hop), seven items on upper-limb coordination (e.g., dribbling a ball), and five items on strength (e.g., sit-ups). Scale scores for the eight subtests (mean, 15; SD, 5) are organized into four motor area composites: fine manual control, manual coordination, body coordination, and strength and agility. By summing up these four motor area composite scores, the total motor composite is calculated. The motor area and total motor composite scores yield a standard score (mean, 50; SD, 10). The BOT-2 also has descriptive categories: well-above average (standard score of 70 and above), above average (standard score of 60 to 69), average (standard score of 41 to 59), below average (standard score of 31 to 40), and well-below average (standard score of 30 or less). In this study, children in below average or well-below average categories were identified as having DCD [27].

The BOT-2 has appropriate reliability and validity in its development study [20]. The internal consistency reliability of the total motor composite was around 0.95. Individuals with DCD showed significantly lower performances in the BOT-2 than the non-clinical group. Cronbach’s alpha reliability coefficient of the BOT-2 total motor composite from the sample of the current study was 0.91.

#### 2.2.2. The Developmental Coordination Disorder Questionnaire 2007 (DCDQ’07)

The DCDQ’07 measures a child’s coordination in daily life to assist in identifying children with DCD aged 5 to 15 years [23,32]. Parents report their child’s motor skills based on comparison with those of the child’s peers. The DCDQ’07 has three factors consisting of 15 items: (1) six items on control during movement (e.g., “Your child throws a ball in a controlled and accurate fashion”), (2) four items on fine motor/handwriting (e.g., “Your child’s printing or writing or drawing in class is fast enough to keep up with the rest of the children in the class”), and (3) five items on general coordination (e.g., “Your child learns new motor tasks easily and does not require more practice or time than other children to achieve the same level of skill”). The child’s motor skill for each item is rated on a 5-point Likert scale (1 = “not at all like your child”; 2 = “a bit like your child”; 3 = “moderately like your child”; 4 = “quite a bit like your child”; and 5 = “extremely like your child”). Scores for three factors are summed to yield a total score. The lower the score, the poorer the motor coordination. If the total score is less than or equal to the cutoff score, the child is classified as “indication of, or suspect for, DCD.” Otherwise, the child is thought to be “probably not DCD.” The cutoff score for identifying DCD differs depending on age, which is 46 or less for children aged 5 years to 7 years 11 months, 55 or less for children aged 8 years to 9 years 11 months, and 57 or less for children aged 10 years to 15 years. These cutoff scores were derived from Canadian and British children [23]. The present study used a Korean translated version of the DCDQ’07 [33].

The DCDQ’07 has good reliability and validity in its development study [23,32]. The internal consistency reliability of the total score was 0.89. The DCDQ’07 discriminated children with and without DCD with a sensitivity of 84.6% and a specificity of 70.8%. Cronbach’s alpha reliability coefficient of the DCDQ’07 total score from the sample of the current study was 0.92.

### 2.3. Data Analysis

Children of this study were divided into two groups consistent with age groups of the DCDQ’07. The first group consisted of children aged between 8 years and 9 years 11 months (the 8 to 9 age group). The second group consisted of children aged between 10 years and 12 years 11 months (the 10 to 12 age group). Descriptive statistics were performed to examine the mean and standard deviation of the BOT-2 and the DCDQ’07. Pearson’s correlation was used to determine the relationship between the BOT-2 (four motor areas and total motor composite scores) and the DCDQ’07 (three factors and total scores). The correlation coefficient could be interpreted as strong (≥0.7), moderate (0.3–0.7), or weak (<0.3) [34].

To determine to what extent the original cutoff score of the DCDQ’07 classified Korean children with or without probable DCD, the sensitivity and specificity of the DCDQ’07 were analyzed using the BOT-2 as a reference. Sensitivity was defined as the proportion of children who were categorized as having an “indication of, or suspect for, DCD” in the DCDQ’07 among children who were categorized as below average or well-below average in the BOT-2 [27,35]. Specificity was defined as the proportion of children who were categorized as “probably not DCD” in the DCDQ’07 among children who were categorized as average, above average, or well-above average in the BOT-2. Preferable sensitivity and specificity for a diagnostic test are 80% and 90%, respectively [32]. For a screening test, the sensitivity is more emphasized than the specificity [36].

The adjusted DCDQ’07 cutoff score for Korean children was estimated using ‘the closest to (0,1) criterion’ with a receiver operating characteristic (ROC) curve [37]. The point that had the shortest distance from the point (0, 1) on the ROC curve was found. The value of area under the curve (AUC) could be considered as outstanding discrimination (≥0.9), excellent discrimination (0.8–0.9), acceptable discrimination (0.7–0.8), poor discrimination (0.5–0.7), or no discrimination (= 0.5) [38,39]. Using the adjusted DCDQ’07 cutoff score, sensitivity and specificity of the DCDQ’07 were recalculated.

All statistical analyses were performed using IBM SPSS Statistics, version 22.0 (IBM Corp., Armonk, NY, USA). The statistical significance level was set at *p* < 0.05.

## 3. Results

Table 1 shows descriptive results of the DCDQ’07 and the BOT-2. The DCDQ’07 total score of Korean children was 58.62 for the 8 to 9 age group and 62.84 for the 10 to 12 age group. The total score was divided by the number of items to calculate the average score. In the 8 to 9 age group, the average score was 3.91 for responses of “moderately like your child (3 point)” and “quite a bit like your child (4 point)”. In the 10 to 12 age group, the average score was 4.19 for “quite a bit like your child (4 point)” and “extremely like your child (5 point)”.

**Figure 1:**
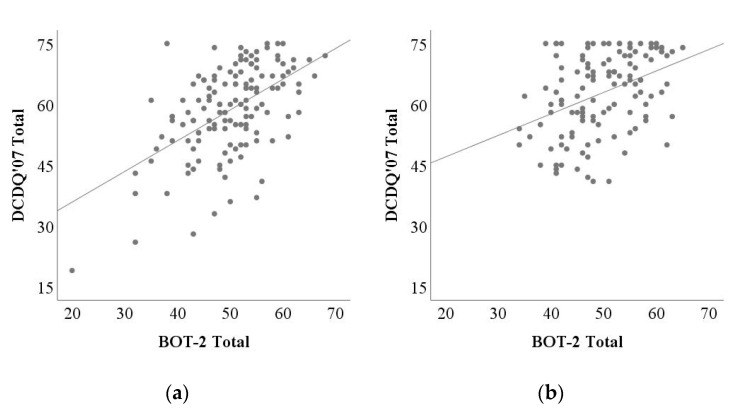
Correlation between DCDQ’07 totals and BOT-2 totals for (**a**) the 8 to 9 age group and (**b**) the 10 to 12 age group. DCDQ’07: Developmental Coordination Disorder Questionnaire 2007; BOT-2: Bruininks-Oseretsky Test of Motor Proficiency Second Edition.

Correlation results between the DCDQ’07 and the BOT-2 are shown in Table 2. Correlation coefficients between DCDQ’07 total scores and BOT-2 total scores were moderate (0.54 for the 8 to 9 age group and 0.40 for the 10 to 12 age group, Figure 1). Strengths of correlations between the three factors of the DCDQ’07 and the four motor areas of the BOT-2 differed depending on the relationship. The control during movement factor of the DCDQ’07 showed moderate correlations with the manual coordination and the strength and agility areas of the BOT-2 in both groups. The fine motor/handwriting factor of the DCDQ’07 was correlated most strongly with the fine manual control area of the BOT-2. The correlation was moderate in the 8 to 9 age group but weak in the 10 to 12 age group. The general coordination factor of the DCDQ’07 was significantly correlated with all motor areas of the BOT-2, which was moderate in the 8 to 9 age group and weak to moderate in the 10 to 12 age group.

For twelve correlations between three DCDQ’07 factors and four BOT-2 motor areas, all results were significant for the 8 to 9 age group. In contrast, for the 10 to 12 age group, significant results were found only for seven correlations. There was no significant correlation between the control during movement factor of the DCDQ’07 and two motor areas (fine manual control and body coordination) of the BOT-2, or between the fine motor/handwriting factor of the DCDQ’07 and three motor areas (manual coordination, body coordination, and strength and agility) of the BOT-2.

The original DCDQ’07 cutoff score was initially used to classify children with or without probable DCD. For the 8 to 9 age group, the cutoff correctly classified 9 of 14 children with probable DCD (sensitivity of 64.3%) and 83 of 120 children without probable DCD (specificity of 69.2%). For the 10 to 12 age group, the cutoff accurately categorized 6 of 11 children with probable DCD (sensitivity of 54.5%) and 81 of 111 children without probable DCD (specificity of 73%).

**Figure 2:**
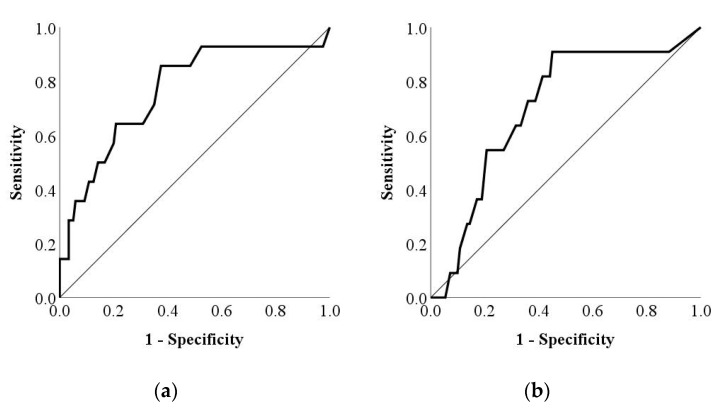
Receiver operating characteristic curve for the DCDQ’07 in (**a**) the 8 to 9 age group (AUC, 0.77) and (**b**) the 10 to 12 age group (AUC, 0.70). DCDQ’07: Developmental Coordination Disorder Questionnaire 2007; AUC: area under the curve.

The adjusted DCDQ’07 cutoff score for each group was estimated using ROC analysis (Figure 2). For the 8 to 9 age group, the adjusted cutoff of 57 showed a sensitivity of 85.7% and a specificity of 62.5%, with an AUC of 0.77. For the 10 to 12 age group, the adjusted cutoff of 60 showed a sensitivity of 72.7% and a specificity of 64%, with an AUC of 0.70.

## 4. Discussion

This study demonstrated that Korean parents’ perception of their children’s motor skills was related to their actual motor proficiency. The DCDQ’07 total score was correlated with the BOT-2 total composite, indicating that Korean parents reported their children’s daily motor skills with general accuracy. The correlation between the DCDQ’07 total score and the BOT-2 total composite in Korean children was comparable to results shown for Brazilian children [27]. The strength of the correlation between the corresponding constructs in the DCDQ’07 and the BOT-2 was more pronounced. The control during movement of the DCDQ’07 was correlated with the manual coordination and the strength and agility of the BOT-2 more strongly than with other subtests of the BOT-2. Likewise, the fine motor/handwriting of the DCDQ’07 most strongly correlated with the fine manual control of the BOT-2. These findings indicate that parents differentially perceived their child’s motor skills and suggest that parental perceptions reported on the DCDQ’07 could validly provide information on their children’s motor skills.

Compared to children in the 8 to 9 age group, those in the 10 to 12 age group had insignificant or weak results for several correlations between DCDQ’07 factors and BOT-2 motor areas. For example, the correlation between the fine motor/handwriting of the DCDQ’07 and the fine manual control of the BOT-2 was weaker in the 10 to 12 age group than in the 8 to 9 age group. This result was consistent with the finding of a previous research [40] showing that parental perception of older children’s movement skills was less correlated with children’s actual motor competence than that of younger children. Motor tasks in daily living are skilled as children age [41], which can lead to higher DCDQ’07 scores for older children. The less differentiated DCDQ’07 score could show a low correlation with the BOT-2 that measures individual differences in detail. Taken together, these results suggest that the DCDQ’07 total score could provide more valid information than its factor score showing a low correlation coefficient, especially in older children.

This study also calculated the sensitivity and specificity of the DCDQ’07 in Korea. When the BOT-2 was used as the standard, the original DCDQ’07 cutoff score accurately classified 54.5–64.3% of the children suspected as having DCD (sensitivity) and 69.2–73% of the children without DCD (specificity). Compared to the sensitivity of 88.5–88.6% and specificity of 66.7–75.6% in the DCDQ’07 development study in Canada and the United Kingdom [23], the sensitivity of the original cutoff score was much lower for Korean children. Since the DCDQ’07 was designed to screen children suspected of having DCDQ, its sensitivity was more important than its specificity [23]. This study estimated adjusted cutoff scores for Korean children using the ROC curve. The adjusted cutoff score showed a sensitivity of 85.7% and a specificity of 62.5% in the 8 to 9 age group and a sensitivity of 72.7% and specificity of 64% in the 10 to 12 age group. The sensitivity and specificity of the adjusted cutoff for the 8 to 9 age group were compatible with those of the DCDQ’07 development study. However, the classification accuracy of the adjusted cutoff for the 10 to 12 age group was not sufficiently high compared to results of the DCDQ’07 development study. Such differences in sensitivity and specificity between studies could be due to characteristics of samples. While this study recruited children only from the community, the DCDQ’07 development study [23] included a clinical sample and a community sample. The sensitivity and specificity for the clinical sample were higher than those for the community sample [42]. The sensitivity and specificity can differ depending on whether the clinical sample is included [43,44]. A psychometric study of the DCDQ’07 for Italian community children reported that its sensitivity and specificity were 58% and 67% for 10 to 12 years old, respectively [43]. In contrast, in another Italian sample aged 5 to 11 years, which included children with DCD as well as typically developing children, the sensitivity was 88% and the specificity was 96% [44]. Moreover, all seven children with DCD and 13 among 14 typically developing children aged 10 to 11 years were correctly classified. These two studies demonstrated that the classification using both clinical and community samples was more accurate than that including community samples alone. Future studies need to include clinical samples to validate the adjusted cutoff scores for Korean children.

DCDQ’07 total scores of all children in the current study (60.63; age range, 8 to 12 years) were comparable to scores reported in a previous study on Korean children (61.75; age range, 5 to 15 years) [45]. These scores in the current study were higher than those for Brazilian children (58.26; age range, 6 to 10 years) [27] but lower than those for Canadian and British children in the original manual (61.79; age range, 5 to 15 years) [32], Turkish children (63.79; age range, 5 to 15 years) [46], and Spanish children (64.64; age range, 6 to 12 years) [47]. Since parents of older children rated their children’s motor skills more positively and age ranges differed slightly across studies, we must carefully compare these scores between studies. These differences in DCDQ’07 total scores across countries raise the possibility that parents’ response tendencies could be affected by their culture.

This study has a limitation that needs further research. This study calculated the sensitivity and specificity of the DCDQ’07 in a community population using the BOT-2 standard score as the criterion. Since the sensitivity and specificity of the DCDQ’07 were found to be higher in a clinical sample than in a community sample [42], future studies need to examine the accuracy of the DCDQ’07 in clinical samples based on a DCD diagnosis made by a psychiatrist.

## 5. Conclusions

This study demonstrated the relationship between Korean parents’ perception of their children’s motor skills measured with the DCDQ’07 and children’s actual motor proficiency assessed with the BOT-2. The DCDQ’07 total score had a moderate correlation with the BOT-2 total composite score. However, there were lower correlations between DCDQ’07 factors and BOT-2 motor areas, especially in the older age group. These results suggest that the DCDQ’07 total score could present better children’s motor skills than its factor score. In addition, this study estimated the adjusted cutoff score of the DCDQ’07 with the BOT-2 as the criterion. The adjusted cutoff score could be considered useful in differentiating younger children with DCD. In contrast, the adjusted cutoff score for older children requires further validation. A future study should include children with DCD to improve the classification accuracy. In conclusion, this study established the validity of the DCDQ’07 to help accurately identify children suspected of having DCD.

## Figures and Tables

**Table 1:** Descriptive results of the DCDQ’07 and the BOT-2, mean (SD).

	8 to 9 Age	10 to 12 Age	Total
DCDQ’07			
Control during movement	23.45 (4.84)	24.96 (4.50)	24.17 (4.74)
Fine motor/handwriting	16.90 (3.31)	17.72 (2.84)	17.29 (3.12)
General coordination	18.28 (4.51)	20.16 (3.93)	19.17 (4.34)
Total	58.62 (11.00)	62.84 (9.80)	60.63 (10.64)
BOT-2			
Fine manual control	52.19 (8.02)	49.58 (6.94)	50.95 (7.62)
Fine motor precision	16.33 (4.41)	15.22 (3.65)	15.80 (4.10)
Fine motor integration	15.54 (3.34)	14.83 (3.54)	15.20 (3.45)
Manual coordination	47.47 (7.83)	47.70 (8.91)	47.58 (8.35)
Manual dexterity	17.43 (3.89)	16.63 (3.76)	17.05 (3.84)
Upper-limb coordination	10.40 (3.90)	11.68 (4.47)	11.01 (4.22)
Body coordination	47.22 (8.03)	49.18 (7.93)	48.16 (8.03)
Bilateral coordination	15.02 (3.68)	16.03 (3.68)	15.50 (3.71)
Balance	13.03 (4.20)	13.41 (4.20)	13.21 (4.20)
Strength and agility	53.75 (8.78)	54.27 (7.08)	54.00 (8.00)
Running speed and agility	17.69 (3.75)	17.86 (3.05)	17.77 (3.43)
Strength	15.04 (4.24)	15.85 (3.72)	15.43 (4.02)
Total motor composite	49.99 (7.87)	49.79 (7.32)	49.89 (7.60)

DCDQ’07: Developmental Coordination Disorder Questionnaire 2007; BOT-2: Bruininks-Oseretsky Test of Motor Proficiency Second Edition. DCDQ’07 results are presented as sums of raw scores. BOT-2 results are presented as scale scores (mean = 15, SD = 5) for eight subtests or as standard scores (mean = 50, SD = 10) for four motor areas and a total motor composite.

**Table 2:** Correlation coefficients between the DCDQ’07 and the BOT-2.

Variables	8 to 9 Age					10 to 12 Age					Total				
	FMC	MC	BC	SA	BOT-2 Total	FMC	MC	BC	SA	BOT-2 Total	FMC	MC	BC	SA	BOT-2 Total
Control during movement	0.32 **	0.45 **	0.36 **	0.44 **	0.51 **	0.03	0.47 **	0.16	0.39 **	0.41 **	0.17 **	0.45 **	0.28 **	0.42 **	0.46 **
Fine motor/handwriting	0.45 **	0.26 **	0.30 **	0.19 *	0.38 **	0.21*	0.14	−0.02	0.03	0.13	0.32 **	0.20 **	0.18 **	0.13 *	0.27 **
General coordination	0.42 **	0.38 **	0.40 **	0.31 **	0.50 **	0.19 *	0.39 **	0.21*	0.30 **	0.42 **	0.28 **	0.38 **	0.34 **	0.31 **	0.45 **
DCDQ’07 total	0.45 **	0.43 **	0.41 **	0.38 **	0.54 **	0.15	0.41 **	0.15	0.31 **	0.40 **	0.28 **	0.41 **	0.31 **	0.35 **	0.47 **

* *p* < 0.05; ** *p* < 0.01; DCDQ’07: Developmental Coordination Disorder Questionnaire 2007; BOT-2: Bruininks-Oseretsky Test of Motor Proficiency Second Edition; FMC: fine manual control; MC: manual coordination; BC: body coordination; SA: strength and agility.

## Data Availability

The data that support the findings of this study are available from the corresponding author upon reasonable request. The data are not publicly available because they concern the privacy of children.
